# Impact of Spread Through Air Spaces on Outcomes After Uniportal VATS Resection for Lung Adenocarcinoma: A Propensity Score–Matched Analysis

**DOI:** 10.3390/jpm16080410

**Published:** 2026-07-30

**Authors:** Michele Salati, Anna Chiara Nanto, Mara Romito, Alberto Roncon, Michela Tiberi, Gian Marco Guiducci, Francesco Xiumè, Francesca Barbisan, Gaia Goteri, Majed Refai

**Affiliations:** 1 Thoracic Surgery Unit, University Hospital of Marche, via Conca 71, 60126 Ancona, Italy; annachiara.nanto@ospedaliriuniti.marche.it (A.C.N.); mara.romito@ospedaliriuniti.marche.it (M.R.); alberto.roncon@ospedaliriuniti.marche.it (A.R.); michela.tiberi@ospedaliriuniti.marche.it (M.T.); gianmarco.guiducci@ospedaliriuniti.marche.it (G.M.G.); francesco.xiume@ospedaliriuniti.marche.it (F.X.); majed.refai@ospedaliriuniti.marche.it (M.R.); 2 Department of Pathology, Polytechnic University of Marche, via Tronto 10/a, 60126 Ancona, Italy; francesca.barbisan@osppedaliriuniti.marche.it (F.B.); gaia.goteri@ospedaliriuniti.marche.it (G.G.)

**Keywords:** lung adenocarcinoma, STAS, spread through air spaces, uniportal VATS, sublobar resection, lobectomy, recurrence, overall survival, personalized surgery

## Abstract

**Background**: Tumor Spread Through Air Spaces (STAS) is a histopathological pattern of invasion associated with aggressive behavior in lung adenocarcinoma. Its prognostic impact and implications for surgical strategy remain controversial. This study evaluated the impact of STAS on survival and recurrence outcomes in patients undergoing uniportal video-assisted thoracoscopic surgery (U-VATS) for lung adenocarcinoma. **Methods**: We retrospectively analyzed consecutive patients with histologically confirmed lung adenocarcinoma who underwent U-VATS resection between January 2020 and January 2023 at a single tertiary referral center. Propensity score matching (1:1) was performed to reduce selection bias between STAS-positive and STAS-negative patients. Overall survival (OS) and recurrence-free survival (RFS) were analyzed using Kaplan–Meier estimates and log-rank tests. Multivariable Cox proportional hazards regression models were used to identify independent predictors of survival. Subgroup analyses were performed according to the type of resection. **Results**: Among 365 included patients, 111 (30.4%) were STAS-positive. After propensity score matching, 222 patients were analyzed (111 STAS-positive and 111 STAS-negative). STAS-positive patients showed a higher overall recurrence rate compared with STAS-negative patients (36.9% vs. 27.9%, *p* = 0.15), with a significantly increased rate of local parenchymal recurrence (18.9% vs. 9.0%, *p* = 0.03). No significant differences in OS or RFS were observed between matched groups. Multivariable Cox regression analysis confirmed that STAS was not independently associated with OS (HR 0.88, 95% CI 0.50–1.53, *p* = 0.643). In STAS-positive patients, sublobar resection was associated with significantly worse survival outcomes compared with lobectomy and emerged as an independent predictor of mortality (HR 2.61, 95% CI 1.14–5.97, *p* = 0.02). **Conclusions**: STAS was associated with an increased risk of local recurrence but was not independently associated with overall survival after U-VATS resection for lung adenocarcinoma. However, in STAS-positive patients, sublobar resection was independently associated with worse survival outcomes, suggesting that surgical extent plays a critical role in this subgroup. These findings support consideration of a personalized surgical approach in patients with STAS-positive lung adenocarcinoma.

## 1. Introduction

Tumor Spread Through Air Spaces (STAS) is a distinct pattern of tumor invasion in lung cancer, characterized by the presence of micropapillary clusters, solid nests, or single tumor cells within the alveolar spaces beyond the edge of the primary tumor [1,2]. Since its introduction in the WHO classification of lung tumors, STAS has emerged as a relevant histopathological feature, particularly in lung adenocarcinoma, where it has been associated with more aggressive tumor behavior.

Growing evidence suggests that STAS is linked to increased recurrence rates and worse oncological outcomes, especially in early-stage non-small cell lung cancer (NSCLC) [3,4,5,6]. Several retrospective studies and meta-analyses have demonstrated that STAS-positive tumors are associated with reduced recurrence-free survival (RFS) and overall survival (OS), although results remain heterogeneous across different patient populations and surgical strategies [7,8].

Notably, the prognostic impact of STAS appears to be strongly influenced by the extent of surgical resection. Sublobar resections, including segmentectomy and wedge resection, have been associated with higher recurrence rates in STAS-positive tumors compared to lobectomy [9,10]. However, this association is not consistently observed across all studies, and the role of surgical margins remains debated [11].

A critical limitation in clinical practice is that STAS can only be reliably identified on postoperative histopathological examination and cannot be accurately diagnosed using preoperative biopsy techniques [12,13]. This limitation significantly restricts its use in preoperative risk stratification and surgical planning.

In the context of personalized medicine, understanding the clinical significance of STAS is essential to refine surgical decision-making and optimize patient-specific treatment strategies.

The aim of this study was to evaluate the impact of STAS on survival and recurrence outcomes in a propensity score-matched cohort of patients undergoing uniportal VATS resection for lung adenocarcinoma. Additionally, we investigated the influence of surgical extent on outcomes in STAS-positive patients.

## 2. Materials and Methods

### 2.1. Study Design and Patients’ Information

This retrospective observational study included consecutive patients aged ≥18 years with histologically confirmed lung adenocarcinoma who underwent uniportal video-assisted thoracoscopic surgery (U-VATS) between January 2020 and January 2023 at a single tertiary referral center (Unit of Thoracic Surgery, Azienda Ospedaliero Universitaria delle Marche, Ancona, Italy). Patients with histological diagnoses other than adenocarcinoma, metastatic disease, incomplete pathological assessment of STAS, incomplete follow-up, and conversions to thoracotomy were excluded from the study. Patients who received neoadjuvant chemotherapy, radiotherapy, immunotherapy, or combined neoadjuvant treatments before surgery were excluded. Moreover, patients with active malignancy or undergoing ongoing oncological treatment at the time of lung resection were not eligible for inclusion in the study.

The study was conducted in accordance with the Declaration of Helsinki and was notified to the Ethics Committee of the Marche Region. Ethical review and approval were not required for this study, as it is a retrospective analysis based on anonymized data collected during routine clinical practice. All patients provided informed consent to the use of anonymized clinical data for scientific purposes.

Demographic characteristics, clinicopathological variables, perioperative data, and follow-up information were prospectively collected in the institutional electronic medical registry and verified through hospital medical records at the time of data extraction to ensure completeness of follow-up. All cases were discussed in a multidisciplinary tumor board including thoracic surgeons, oncologists, pulmonologists, radiologists, and pathologists. Functional evaluation was performed according to international recommendations [14].

Surgical procedures included lobectomy, segmentectomy, or wedge resection, all performed via a uniportal VATS approach, as described elsewhere [15]. The extent of pulmonary resection (lobectomy, segmentectomy, or wedge resection) was determined according to current international guidelines, taking into account tumour characteristics, pulmonary functional reserve, patient comorbidities, and multidisciplinary clinical judgment. Systematic lymph node dissection or sampling was performed according to the recommendations of the European Society of Thoracic Surgeons (ESTS) [16]. At least two mediastinal lymph node stations, together with hilar, lobar, and interlobar lymph nodes, were routinely sampled or dissected in all patients to ensure accurate pathological staging. All resections achieved microscopically negative surgical margins (R0). For anatomical segmentectomies, surgical margins were planned according to accepted oncological principles. Segmentectomies were performed with a margin distance of at least 2 cm. In patients undergoing wedge resection, margin adequacy was confirmed on the final pathological specimen.

Operative and postoperative patient management was standardized using the institutional Enhanced Pathways of Care (EPC) protocol, as previously described [15]. All surgical specimens were examined on permanent formalin-fixed, paraffin-embedded sections by dedicated thoracic pathologists according to routine institutional practice. To minimize the risk of artifactual tumour cell displacement, a clean knife was routinely used during specimen sectioning, in accordance with institutional pathological practice. Histopathological evaluation was performed following the criteria of the World Health Organization (WHO) Classification of Thoracic Tumours [1,17].

Spread Through Air Spaces (STAS) was defined as the presence of micropapillary clusters, solid nests, or isolated tumour cells beyond the edge of the main tumour within the surrounding alveolar spaces [9]. STAS assessment was performed on the definitive surgical specimen according to the institutional standardized pathological protocol adopted since January 2020.

All histopathological variables, including tumour size, histological subtype, pathological T stage, pathological N stage, lymphovascular invasion, vascular invasion, and STAS status, were retrieved from the final pathology reports. 

### 2.2. Follow Up Program and Outcomes

Postoperative follow-up was performed according to the institutional surveillance protocol and current international guidelines [18]. Patients underwent chest computed tomography every six months during the first two postoperative years and annually thereafter up to five years. Additional diagnostic investigations were performed when clinically indicated.

Clinical outcomes were prospectively collected and included both survival and recurrence-related endpoints. Overall survival (OS) was defined as the time interval between the date of surgery and death from any cause or last follow-up. Recurrence-free survival (RFS) was defined as the time from surgery to the first documented evidence of disease recurrence or last follow-up.

When recurrence occurred, its pattern was carefully categorized as local (parenchymal), regional (lymph nodal), or distant metastatic disease, based on radiological and/or histological confirmation. This classification allowed for a more detailed assessment of recurrence dynamics in relation to STAS status. For recurrence-free survival analyses, recurrence was considered the event of interest, whereas patients who died without documented recurrence were censored at the time of death.

The last follow-up update and data censoring were performed in December 2024. Median follow-up duration was 38.9 months (IQR 30.5–49.7).

### 2.3. Statistical Analysis

Data for the present analysis were retrieved from a prospectively maintained institutional database. Prior to analysis, all variables underwent data quality assessment and cleaning procedures. Variables with a completeness rate below 90%, as well as patients with missing data on primary outcomes, were excluded. For variables with less than 10% missing data, single mean imputation was applied before propensity score estimation. Missing values were limited to BMI, FEV1, and DLCO, with completeness rates of 98.8%, 98.3%, and 97.5%, respectively (corresponding to missing rates of 1.2%, 1.7%, and 2.5%). Given the very low proportion of missing values, this approach was considered appropriate to preserve the complete study cohort while minimizing the potential impact of missing data on the propensity score estimation. Continuous variables were tested for normality using the Shapiro–Wilk test. Normally distributed variables were compared using the unpaired Student’s *t*-test, whereas non-normally distributed variables were analyzed using the Mann–Whitney *U* test. Categorical variables were compared using the chi-square test, ensuring that all expected cell counts were adequate.

To reduce the effect of potential confounding factors, propensity score matching (PSM) was performed between STAS-positive and STAS-negative patients. The propensity score model included variables considered clinically relevant and potentially associated with both STAS presence and outcomes: age, sex, American Society of Anesthesiologists (ASA) score, body mass index (BMI), forced expiratory volume in 1 s (FEV1), carbon monoxide diffusing capacity (DLCO), history of coronary artery disease, arterial vascular disease, cerebrovascular disease, chronic kidney disease, chronic liver disease, diabetes, previous neoplastic disease, pathological T stage, pathological N stage, and type of surgical resection (lobectomy, segmentectomy, or wedge resection). Previous neoplastic disease was defined as any prior malignancy treated with curative intent and without evidence of active disease at the time of lung resection.

To minimize baseline differences between STAS-positive and STAS-negative patients, propensity score matching (PSM) was performed using a 1:1 nearest-neighbour algorithm without replacement. Propensity scores were estimated by logistic regression including clinically relevant demographic, functional, pathological, and surgical variables. A caliper width equal to 0.2 standard deviations of the logit of the propensity score was applied. Covariate balance after matching was assessed using standardized mean differences (SMDs), with values < 0.10 considered indicative of adequate balance.

Outcomes were compared between matched groups in terms of recurrence and mortality rates. Additional subgroup analyses were performed according to the type of surgical resection.

Continuous variables were presented as mean ± standard deviation (SD) or median with interquartile range (IQR), as appropriate, whereas categorical variables were reported as frequencies and percentages. Between-group comparisons were performed using Student’s *t*-test or the Mann–Whitney U test for continuous variables and the χ^2^ test or Fisher’s exact test for categorical variables, as appropriate.

Overall survival (OS) and recurrence-free survival (RFS) were estimated using the Kaplan–Meier method and compared using the log-rank test. Variables considered clinically relevant or associated with survival in univariable analyses were entered into multivariable Cox proportional hazards regression models. Variables included in the multivariable Cox regression model were selected a priori according to their established prognostic relevance for overall survival and were not intended to replicate the full set of variables used for propensity score matching. Hazard ratios (HRs) with 95% confidence intervals (CIs) were reported. The proportional hazards assumption was assessed using Schoenfeld residuals.

A two-sided *p* value < 0.05 was considered statistically significant. All analyses were performed using Stata version 14.0 (StataCorp, College Station, TX, USA).

## 3. Results

A total of 365 patients with histologically confirmed lung adenocarcinoma who underwent uniportal VATS resection were included in the initial cohort. Among them, 111 patients (30.4%) were STAS-positive and 254 (69.6%) were STAS-negative. The mean age of the study population was 69.1 ± 9.6 years, and 55.6% of patients were male (Table 1).

The most frequently performed procedure was lobectomy (65.7%), followed by wedge resection (24.4%) and segmentectomy (9.9%). Systematic lymph node dissection was performed in all patients. No intraoperative conversions from uniportal video-assisted thoracoscopic surgery (U-VATS) to thoracotomy occurred among the patients included in the study.

Before propensity score matching, baseline characteristics were generally comparable between the two groups, with the exception of a higher prevalence of chronic liver disease in STAS-positive patients.

### 3.1. Propensity Score Matching and Overall Outcomes

Propensity score matching generated 111 well-balanced pairs (n = 222 patients). After matching, no clinically meaningful differences were observed between STAS-positive and STAS-negative groups across demographic variables, comorbidities, pulmonary function, pathological staging, or type of surgical resection (Table 2).

Standardized mean differences were below 0.1 for all included variables, confirming adequate covariate balance.

To provide an overall view of oncological outcomes in the matched cohort, recurrence patterns and mortality rates are summarized in Table 3. Overall, recurrence occurred in 32.4% of patients. Although STAS-positive patients showed a higher recurrence rate compared with STAS-negative patients (36.9% vs. 27.9%), this difference did not reach statistical significance (*p* = 0.15). Similarly, overall mortality rates were comparable between the two groups (27.0% vs. 24.3%, *p* = 0.64). Among the 57 deaths recorded during follow-up, 36 were cancer-related and 21 were attributable to other causes. Overall survival analyses were performed using all-cause mortality as the predefined endpoint. 

However, when analyzing recurrence patterns in detail, a significant difference emerged in local (parenchymal) recurrence, which was more than twice as frequent in STAS-positive patients compared with STAS-negative patients (18.9% vs. 9.0%, *p* = 0.03). No statistically significant differences were observed in nodal or distant recurrence.

### 3.2. Survival Analysis

**Figure 1:**
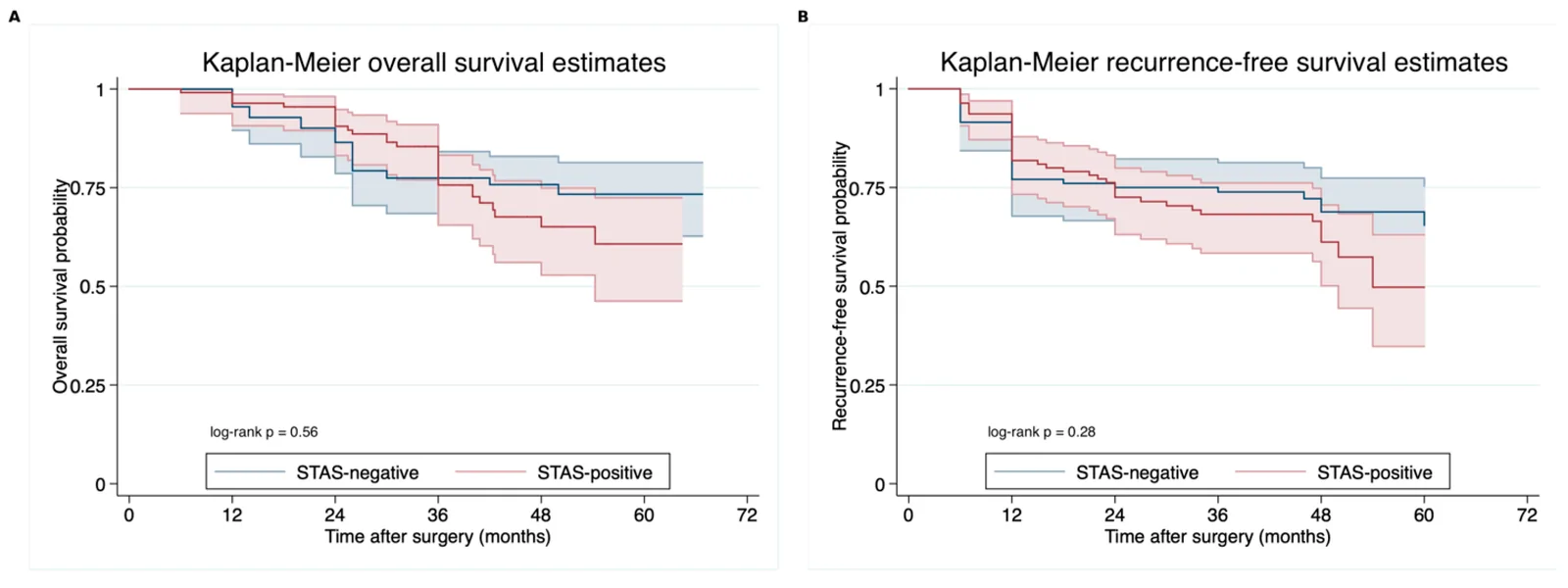
Overall survival (OS) and recurrence-free survival (RFS) after propensity score matching. (**A**) Overall survival (OS) in the matched cohort (n = 222). No statistically significant difference was observed between STAS-positive and STAS-negative patients (log-rank *p* = 0.56). (**B**) Recurrence-free survival (RFS) in the matched cohort (n = 222). Recurrence-free survival did not differ significantly between the two groups (log-rank *p* = 0.28). Blue curves represent STAS-negative patients and red curves represent STAS-positive patients. Shaded areas indicate 95% confidence intervals.

In the matched cohort, Kaplan–Meier survival analysis showed no significant differences in overall survival (OS) between STAS-positive and STAS-negative patients. Similarly, recurrence-free survival curves were largely overlapping between the two groups, confirming the absence of a significant association between STAS status and RFS (Figure 1).

The estimated 3-year overall survival was 76.9% in the overall matched cohort, corresponding to 77.4% in STAS-negative patients and 75.7% in STAS-positive patients, consistent with the absence of a significant difference between groups. Likewise, the estimated 3-year recurrence-free survival was 73.5% overall, corresponding to 74.2% in STAS-negative patients and 72.7% in STAS-positive patients.

These findings were confirmed by multivariable Cox regression analysis, which showed that STAS was not independently associated with overall survival (HR 0.88, 95% CI 0.50–1.53, *p* = 0.643). In contrast, increasing age (HR 1.04, 95% CI 1.01–1.07, *p* = 0.022), pathological nodal involvement (HR 2.00, 95% CI 1.42–2.82, *p* < 0.001), and reduced DLCO (HR 0.96, 95% CI 0.94–0.98, *p* < 0.001) were identified as independent predictors of worse survival (Table 4).

Exploratory univariable Cox analyses evaluating additional clinicopathological variables (ever smoking, vascular invasion, and lymphovascular invasion) are reported in Supplementary Table S1. Vascular invasion was associated with worse overall survival in the univariable analysis, whereas ever smoking and lymphovascular invasion were not significantly associated with survival.

### 3.3. Subgroup Analysis: Impact of Surgical Resection in STAS-Positive Patients

A subgroup analysis was conducted focusing on STAS-positive patients to evaluate the impact of the extent of resection on oncological outcomes. All resections achieved microscopically negative surgical margins (R0). Among the patients undergoing wedge resection, the pathological tumour-to-margin distance, available for 25 patients, had a mean of 1.52 cm, a median of 1.40 cm, and a range of 0.70–3.60 cm. Baseline characteristics were generally comparable between the two surgical groups, with the exception of DLCO, which was significantly lower among patients undergoing sublobar resection. Detailed baseline characteristics of the STAS-positive subgroup are provided in Supplementary Table S2.

Patients who underwent sublobar resection demonstrated significantly worse outcomes compared with those treated with lobectomy (Table 5). In particular, recurrence rates were markedly higher in the sublobar group (50% vs. 29.6%, *p* = 0.03), with a notable increase in local recurrence (30% vs. 12.7%, *p* = 0.02). Mortality was also significantly higher in patients undergoing sublobar resection (40% vs. 19.7%, *p* = 0.02).

**Figure 2:**
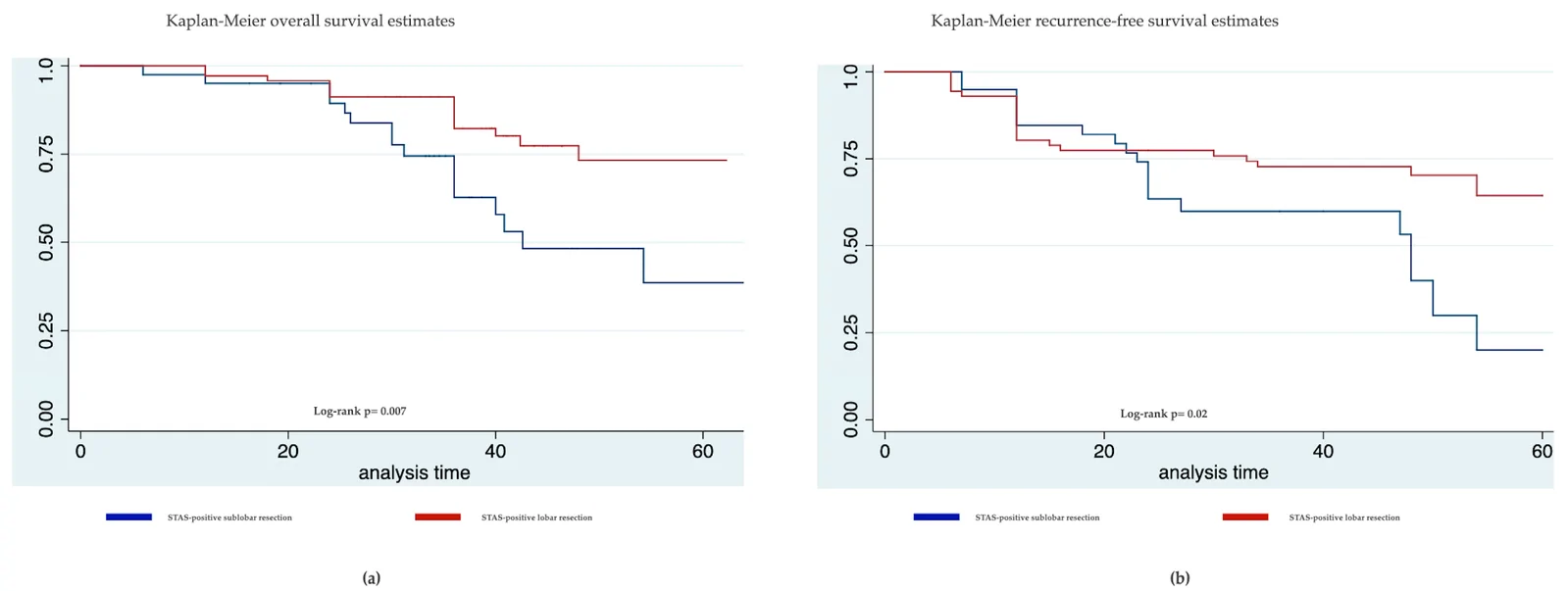
Overall survival (OS) and recurrence-free survival (RFS) in STAS-positive according to surgical extent. (**a**). Overall survival in STAS-positive patients according to surgical procedure. Kaplan–Meier curves comparing overall survival (OS) between STAS-positive patients undergoing lobectomy and those undergoing sublobar resection (segmentectomy or wedge resection). A significantly worse survival was observed in the sublobar group (log-rank *p* = 0.007). (**b**). Recurrence-free survival in STAS-positive patients according to surgical procedure. Kaplan–Meier curves comparing recurrence-free survival (RFS) between STAS-positive patients treated with lobectomy and those undergoing sublobar resection. Sublobar resection was associated with significantly worse RFS (log-rank *p* = 0.02).

Kaplan–Meier analysis confirmed these findings (Figure 2), showing significantly worse overall survival and recurrence-free survival in STAS-positive patients treated with sublobar resection compared with lobectomy.

In STAS-positive patients, multivariable Cox regression analysis (Table 6) identified the type of surgical resection as an independent predictor of overall survival. In particular, sublobar resection was associated with a significantly increased risk of mortality compared with lobectomy (HR 2.61, 95% CI 1.14–5.97, *p* = 0.023).

Pathological nodal involvement (pN) also emerged as a strong adverse prognostic factor (HR 2.31, 95% CI 1.42–3.76, *p* = 0.001), whereas higher DLCO was associated with improved survival (HR 0.96, 95% CI 0.93–0.99, *p* = 0.003).

Age and pathological T stage were not significantly associated with overall survival in this subgroup.

## 4. Discussion

In this propensity score-matched analysis of patients undergoing uniportal VATS resection for lung adenocarcinoma, we investigated the prognostic impact of Tumor Spread Through Air Spaces (STAS) on survival and recurrence outcomes.

Our findings can be summarized as follows: STAS was not independently associated with overall survival in the matched cohort; STAS-positive tumors showed a significantly higher rate of local (parenchymal) recurrence; and, among STAS-positive patients, sublobar resection was associated with less favourable oncological outcomes than lobectomy. Although these subgroup findings should be interpreted cautiously, they support the hypothesis that the prognostic impact of STAS may vary according to the extent of pulmonary resection.

The prognostic role of STAS in NSCLC has been increasingly investigated over the last decade, although available evidence remains partially heterogeneous. Since the first description by Kadota et al. [8], STAS has been considered a potential marker of aggressive tumor biology, particularly in lung adenocarcinoma. Consistent with this biological rationale, several retrospective studies have subsequently reported associations between STAS and increased recurrence rates, reduced recurrence-free survival, and worse overall survival [4,5,6,7].

A large meta-analysis by Chen et al. [19], including more than 4000 NSCLC patients, showed that STAS was associated with a significantly increased risk of recurrence and poorer long-term survival, although substantial heterogeneity persisted among the included studies. 

The prognostic impact of STAS appears to be particularly relevant in lung adenocarcinoma, where it has consistently been associated with more aggressive tumour behaviour and an increased risk of recurrence. Similarly, Yang et al. [20], focusing specifically on stage I lung adenocarcinoma, reported the negative impact of STAS on long-term oncological outcomes, further supporting its role as a marker of biologically aggressive disease.

However, not all studies have reported a consistent independent effect of STAS on survival. More recent investigations suggested that the prognostic impact of STAS may depend on tumor stage, surgical strategy, and patient selection. In particular, Huang and Petersen [21] reported that the adverse prognostic effect of STAS was more evident after sublobar resection than after lobectomy, suggesting that the interaction between tumour biology and surgical strategy may play a relevant role in determining long-term oncological outcomes. Consistent with these observations, in our propensity score-matched cohort, STAS-positive patients experienced a significantly higher incidence of local (parenchymal) recurrence but STAS was not independently associated with overall survival after multivariable adjustment. Instead, pathological nodal involvement, increasing age, and reduced DLCO emerged as the strongest independent predictors of prognosis. These findings support the hypothesis that the biological aggressiveness associated with STAS may primarily influence local disease control, whereas overall survival appears to be more strongly determined by established prognostic factors, including nodal status and baseline functional reserve.

Nevertheless, our study demonstrated a significantly higher rate of local (parenchymal) recurrence in STAS-positive patients. This finding is particularly noteworthy because it supports the concept that STAS may predominantly influence local disease control, even in the absence of an independent association with overall survival. Kadota et al. [8] previously demonstrated that STAS-positive tumors treated with limited resection showed a higher incidence of locoregional recurrence, suggesting that tumor cells spreading within alveolar spaces may remain beyond the surgical margin after parenchyma-sparing procedures. Similarly, Warth et al. [22] reported that intra-alveolar tumor spread was associated with increased local recurrence and poorer prognosis in pulmonary adenocarcinoma. Taken together, these observations support the biological plausibility of STAS as a marker of local tumour dissemination. However, the exact mechanisms underlying this phenomenon remain incompletely understood and are likely to reflect both intrinsic tumour biology and its interaction with the extent of pulmonary resection. These findings also suggest that the prognostic implications of STAS cannot be interpreted independently of the surgical context in which the tumour is treated.

One of the most clinically significant findings of the present study concerns the impact of surgical extent in STAS-positive patients. We observed significantly higher recurrence and mortality rates in patients undergoing sublobar resection compared with lobectomy. These findings were further supported by multivariable Cox regression analysis, in which sublobar resection remained independently associated with worse overall survival within the STAS-positive subgroup. Because treatment allocation was not randomized, residual treatment-selection bias cannot be excluded despite multivariable adjustment and propensity score matching. Therefore, these findings should be interpreted as demonstrating an association rather than a causal effect of the extent of resection. Furthermore, the separate comparison between segmentectomy and wedge resection was performed as an exploratory, unadjusted analysis. Given the small number of patients in each subgroup, the observed differences should be interpreted with caution, as they may reflect residual case-mix differences and limited statistical power rather than true procedure-specific effects. Our observations are consistent with those reported by Huang and Petersen [23], who found that the adverse prognostic impact of STAS was predominantly observed after thoracoscopic sublobar resection, whereas outcomes following lobectomy were substantially less affected. These findings should, however, be interpreted within the context of contemporary evidence supporting sublobar resection for carefully selected patients with early-stage NSCLC. In particular, the JCOG0802/WJOG4607L trial demonstrated that anatomical segmentectomy provides oncological outcomes at least comparable to lobectomy in peripheral small-sized NSCLC [24]. Our findings do not contradict this evidence but rather suggest that STAS may identify a subgroup of patients in whom the prognostic implications of the extent of pulmonary resection deserve further investigation. 

Previous propensity score-matched and observational studies have reported comparable findings. Eguchi et al. [9] demonstrated that STAS-positive tumors treated with sublobar resection had significantly worse recurrence-free survival and a higher risk of locoregional recurrence than those undergoing lobectomy. Similarly, Huang and Petersen [23] reported inferior survival and recurrence outcomes following thoracoscopic segmentectomy in STAS-positive patients. These observations were further supported by the meta-analysis of Li et al. [11], which demonstrated superior oncological outcomes after lobectomy compared with sublobar resection in stage I STAS-positive NSCLC. 

From a surgical perspective, these findings raise important questions regarding the adequacy of parenchyma-sparing procedures in biologically aggressive tumors. Current international guidelines for sublobar resection are primarily based on tumor size and technical margin criteria [24], without considering STAS status. STAS may represent a pattern of microscopic tumour spread that is not fully captured by conventional margin-based surgical criteria. This issue becomes particularly relevant in the modern era of thoracic surgery, where segmentectomy is increasingly adopted following randomized trials demonstrating non-inferiority compared with lobectomy in selected early-stage NSCLC patients. Our findings suggest that STAS may represent one of the factors deserving further investigation when tailoring the extent of pulmonary resection in patients with early-stage lung adenocarcinoma.

However, the inability to reliably predict STAS preoperatively remains a major limitation for individualized surgical planning. Several recent studies explored radiological and radiomics-based models for STAS prediction [25], evaluating features such as solid component size, tumor density, and textural heterogeneity on CT imaging. However, despite promising preliminary results, no predictive model has yet achieved sufficient accuracy or external validation to support routine clinical implementation. Consequently, although our findings suggest that the extent of pulmonary resection may influence outcomes in patients with STAS-positive tumours, they should not be interpreted as supporting routine modification of the initial surgical strategy based solely on the possibility of occult STAS. Rather, they emphasize the need for more reliable preoperative or intraoperative methods to identify STAS before individualized surgical decision-making can be considered.

In the context of personalized medicine, our findings suggest that STAS may serve as an important postoperative risk stratification marker following surgical resection. Although STAS was associated with an increased risk of local recurrence and less favourable outcomes after sublobar resection, its predominantly postoperative diagnosis currently limits its direct application in preoperative surgical decision-making. Consequently, rather than supporting routine modification of the initial surgical strategy, our findings reinforce the need to develop reliable preoperative or intraoperative approaches for STAS identification, which could eventually contribute to more individualized surgical planning.

Furthermore, patients with STAS-positive tumours may benefit from closer postoperative surveillance, whereas the potential role of tailored adjuvant treatment strategies remains to be clarified in prospective studies. Therefore, improved integration of pathological, radiological, and molecular markers may be necessary to optimize individualized treatment strategies in lung adenocarcinoma.

### Limitations of the Study

Several limitations of this study should be acknowledged. First, its retrospective single-center design inherently exposes the study to potential selection bias, despite the use of propensity score matching to improve baseline comparability between the study groups. Second, although the overall cohort was adequately sized, the relatively small number of patients included in the STAS-positive subgroup analyses may have limited statistical power and reduced the precision of some estimates. In particular, because only 30 deaths occurred within the STAS-positive subgroup, the corresponding multivariable Cox regression analysis should be considered exploratory and interpreted with caution, as the limited number of events may have increased the risk of model instability and overfitting. Third, although all pathological evaluations were performed on definitive surgical specimens by dedicated thoracic pathologists according to a standardized institutional protocol, no formal assessment of interobserver agreement was performed. Fourth, because STAS was diagnosed on permanent pathological specimens, the present study cannot determine whether knowledge of STAS status at the time of surgery would have altered the optimal surgical strategy. 

Moreover, the propensity score model included pathological T and N stage to achieve balanced comparison between the matched groups. This approach was intentionally selected to compare patients with similar pathological disease burden and thereby evaluate the prognostic contribution of STAS within comparable pathological stages. Nevertheless, alternative propensity score specifications excluding postoperative pathological variables are methodologically justifiable and might have yielded different estimates of the independent prognostic effect of STAS. Therefore, our findings should be interpreted within the context of this predefined analytical framework. Finally, although the median follow-up exceeded three years, longer follow-up will be necessary to fully evaluate long-term oncological outcomes, particularly in patients with early-stage disease.

Despite these limitations, the use of propensity score matching, complete follow-up, standardized pathological assessment, and a homogeneous surgical approach strengthen the internal validity of the present findings.

## 5. Conclusions

This propensity score-matched analysis showed that STAS was not independently associated with overall survival but was associated with a significantly increased risk of local recurrence.

Among patients with STAS-positive tumours, sublobar resection was associated with less favourable oncological outcomes than lobectomy, suggesting that the prognostic implications of STAS may vary according to the extent of pulmonary resection.

Although these findings should not be interpreted as supporting routine modification of the initial surgical strategy, they highlight the potential value of STAS as a postoperative marker for risk stratification and emphasize the need for reliable preoperative or intraoperative methods for STAS identification to support more individualized surgical decision-making.

## Figures and Tables

**Table 1:** Baseline characteristics comparison between STAS-positive and STAS-negative patients before matching.

Variable	STAS+ (111)	STAS− (254)	*p*-Value
AGE, y, mean (SD)	69.6 (9.2)	68.9 (9.8)	0.6
SEX, Male, n (%)	60 (54.1%)	143 (56.3%)	0.7
ASA, mean (SD)	1.7 (0.78)	1.7 (0.61)	0.7
BMI, mean (SD)	25.2 (4.1)	26.1 (4.3)	0.06
FEV1, mean (SD)	90.3 (21.1)	92.1 (19.1)	0.4
DLCO, mean (SD)	70.4 (15.9)	71.5 (16.4)	0.6
CAD, yes, n (%)	14 (12.6%)	31 (12.2%)	0.9
AD, yes, n (%)	10 (9%)	21 (8.3%)	0.8
CVD, yes, n (%)	7 (6.3%)	23 (9.1%)	0.4
CKD, yes, n (%)	17 (15.3%)	24 (9.4%)	0.1
CLD, yes, n (%)	19 (17.1%)	6 (2.4%)	<0.001
DIA, yes, n (%)	19 (17.1%)	38 (15.0%)	0.6
PND, yes, n (%)	28 (25.2%)	72 (28.3%)	0.5
pT, n (%)			0.9
1	67 (60.4%)	147 (57.9%)	
2	31 (27.9%)	76 (29.9%)	
3	9 (8.1%)	22 (8.7%)	
4	4 (3.6%)	9 (3.5%)	
pN, n (%)			0.1
0	80 (72.1%)	205 (80.7%)	
1	16 (14.4%)	22 (8.7%)	
2	15 (13.5%)	27 (10.6%)	
TYPE RESECT, n (%)			0.6
Lobectomy	71 (63.9%)	167 (65.7%)	
Segmentectomy	14 (12.6%)	22 (8.7%)	
Wedge Resection	26 (23.4%)	65 (25.6%)	

Values are mean (SD), No. (%), or as otherwise indicated. ASA: American Society of Anesthesiologists, BMI: body mass index, FEV1: forced expiratory volume in 1 s, DLCO: carbon monoxide lung diffusion capacity, CAD: coronary artery disease, AD: arterial vascular disease, CVD: cerebrovascular disease, CKD: chronic kidney disease, CLD: chronic liver disease, DIA: diabetes, PND: previous neoplastic disease, pT: pathologic T stage, pN: pathologic N stage, TYPE RESECT: type of resection.

**Table 2:** Baseline characteristics comparison between the 111 pairs of STAS-positive and STAS-negative patients after matching.

Variable	STAS+ (111)	STAS− (111)	*p*-Value	SMD
AGE, y, mean (SD)	69.6 (9.2)	68.5 (9.1)	0.06	0.1
SEX, Male, n (%)	60 (54.1%)	66 (59.5%)	0.4	0.1
ASA, mean (SD)	1.7 (0.78)	1.6 (0.77)	0.2	0.1
BMI, mean (SD)	25.2 (4.1)	25.0 (3.4)	0.7	0.05
FEV1, mean (SD)	90.3 (21.1)	90.5 (18.5)	0.9	0.01
DLCO, mean (SD)	70.4 (14.9)	72.1 (14.5)	0.07	0.1
CAD, yes, n (%)	14 (12.6%)	13 (11.7%)	0.8	0.03
AD, yes, n (%)	10 (9.0%)	7 (6.3%)	0.4	0.1
CVD, yes, n (%)	7 (6.3%)	6 (5.4%)	0.8	0.04
CKD, yes, n (%)	17 (15.3%)	13 (11.7%)	0.4	0.1
CLD, yes, n (%)	19 (17.1%)	20 (18.0%)	0.9	0.02
DIA, yes, n (%)	19 (17.1%)	14 (12.6%)	0.2	0.1
PND, yes, n (%)	28 (25.2%)	27 (24.3%)	0.9	0.02
pT, n (%)			0.6	0.1
1	67 (60.4%)	60 (54.1%)		
2	31 (27.9%)	38 (34.2%)		
3	9 (8.1%)	11 (9.9%)		
4	4 (3.6%)	2 (1.8%)		
pN, n (%)			0.3	0.08
0	80 (72.1%)	76 (68.5%)		
1	16 (14.4%)	15 (13.5)		
2	15 (13.5%)	20 (18.0%)		
TYPE RESECT, n (%)			0.2	0.09
Lobectomy	71 (63.9%)	80 (72.1%)		
Segmentectomy	14 (12.6%)	12 (10.8%)		
Wedge Resection	26 (23.4%)	19 (17.1%)		

Values are mean (SD), No. (%), or as otherwise indicated. ASA: American Society of Anesthesiologists, BMI: body mass index, FEV1: forced expiratory volume in 1 s, DLCO: carbon monoxide lung diffusion capacity, CAD: coronary artery disease, AD: arterial vascular disease, CVD: cerebrovascular disease, CKD: chronic kidney disease, CLD: chronic liver disease, DIA: diabetes, PND: previous neoplastic disease, pT: pathologic T stage, pN: pathologic N stage, TYPE RESECT: type of resection.

**Table 3:** Specific recurrence and mortality rates comparison.

Cohort	Recurrence	*p* Value	Parenchymal Recurrence	*p* Value	Lymphonodal Recurrence	*p* Value	Distant Recurrence	*p* Value	Death	*p* Value
Overall (n = 222)	72 (32.4%)		31 (13.9%)		30 (13.5%)		67 (30.2%)		57 (25.7%)	
STAS-positive (n = 111)	41 (36.9%)	0.15	21 (18.9%)	0.03	16 (14.4%)	0.69	34 (30.6%)	0.88	30 (27.0%)	0.64
STAS-negative (n = 111)	31 (27.9%)		10 (9.0%)		14 (12.6%)		33 (29.7%)		27 (24.3%)	

Values are presented as n (%). Parenchymal recurrence refers to recurrence within the ipsilateral residual lung parenchyma.

**Table 4:** Multivariable Cox proportional hazards regression analysis for overall survival.

Variable	HR	95% CI	*p*-Value
STAS	0.88	0.50–1.53	0.64
Age	1.04	1.01–1.07	0.02
Sex	0.89	0.51–1.56	0.69
ASA	1.66	0.98–2.80	0.06
Pathological T stage	1.18	0.84–1.65	0.33
Pathological N stage	2.00	1.42–2.82	<0.001
Type of resection	1.59	0.88–2.86	0.12
FEV1	0.98	0.97–1.01	0.09
DLCO	0.96	0.94–0.98	<0.001
DIA	0.73	0.32–1.66	0.45

ASA: American Society of Anesthesiologists, FEV1: forced expiratory volume in 1 s, DLCO: carbon monoxide lung diffusion capacity, DIA: diabetes.

**Table 5:** Oncological outcomes in STAS-positive patients according to the extent of surgical resection.

**A. Primary comparison: Lobectomy versus sublobar resection**										
Cohort	Recurrence	*p* value	Parenchymal recurrence	*p* value	Lymphonodal recurrence	*p* value	Distant recurrence	*p* value	Death	*p* value
STAS-positive LOBECTOMY (n = 71)	21 (29.6%)	0.03	9 (12.7%)	0.02	9 (12.7%)	0.49	19 (26.8%)	0.24	14 (19.7%)	0.02
STAS-positive SUBLOBAR RESECTION (n = 40)	20 (50.0%)		12 (30.0%)		7 (17.5%)		15 (37.5%)		16 (40.0%)	
**B. Exploratory analysis: Outcomes according to individual sublobar procedures**										
Cohort	Recurrence	*p* value	Parenchymal recurrence	*p* value	Lymphonodal recurrence	*p* value	Distant recurrence	*p* value	Death	*p* value
STAS-positive LOBECTOMY (n = 71)	21 (29.6%)	0.01	9 (12.7%)	0.03	9 (12.7%)	0.27	19 (26.8%)	0.43	14 (19.7%)	0.04
STAS-positive SEGMENTECTOMY (n = 14)	10 (71.4%)		6 (42.7%)		4 (28.6%)		6 (42.7%)		7 (50.0%)	
STAS-positive WEDGE RES (n = 26)	10 (38.5%)		6 (23.1%)		3 (11.5%)		9 (34.6%)		9 (34.6%)	

**Table 6:** Multivariable Cox regression analysis for overall survival in STAS-positive patients.

Variable	HR	95% CI	*p*-Value
Age	1.03	0.98–1.07	0.22
Sex (male vs. female)	0.63	0.19–2.01	0.43
ASA	0.90	0.41–1.97	0.80
Pathological T stage	1.15	0.75–1.77	0.52
Pathological N stage	2.31	1.42–3.76	0.001
Type of resection (sublobar vs. lobectomy)	2.61	1.14–5.97	0.02
FEV1	0.98	0.95–1.00	0.06
DLCO	0.96	0.93–0.99	0.003
DIA	0.73	0.22–2.49	0.62

ASA: American Society of Anesthesiologists, FEV1: forced expiratory volume in 1 s, DLCO: carbon monoxide lung diffusion capacity, DIA: diabetes.

## Data Availability

The data supporting the findings of this study are derived from institutional clinical records and are not publicly available due to privacy and ethical restrictions. The data presented in this study are available on reasonable request from the corresponding author.

## Supplementary Materials

The following supporting information can be downloaded at: https://www.mdpi.com/article/10.3390/jpm16080410/s1, Table S1: Exploratory univariable Cox proportional hazards analyses of additional clinicopathological variables not included in the primary propensity score-matching model; Table S2: Baseline characteristics of STAS-positive patients undergoing lobectomy or sublobar resection.
