# Supplementary material for: Impact of Spread Through Air Spaces on Outcomes After Uniportal VATS Resection for Lung Adenocarcinoma: A Propensity Score–Matched Analysis
Source: J Pers Med. 2026 Jul 30;16(8):410. doi: 10.3390/jpm16080410 (PMC13514796; doi:10.3390/jpm16080410)
Supplement: Supplementary file 1 [file jpm-16-00410-s001.zip › jpm-4351598-supplementary.pdf]

Supplementary Table S1. Exploratory univariable Cox proportional hazards analyses of additional clinicopathological variables not included in the primary propensity score-matching model.

Variable	Hazard Ratio (HR)	95% Confidence Interval	p-value
Ever smoking	1.21	0.65–2.25	0.546
Vascular invasion (VI)	2.39	1.08–5.28	0.031
Lymphovascular invasion (LVI)	0.60	0.26–1.39	0.233

Footnote

Exploratory univariable Cox proportional hazards analyses were performed in response to reviewer comments. These variables were intentionally not included in the primary propensity score-matching model, which was prespecified using baseline characteristics considered relevant to treatment allocation. Smoking history was evaluated separately because it was not part of the original matching strategy. Vascular invasion (VI) and lymphovascular invasion (LVI) were analyzed only exploratorily because they are postoperative pathological findings unavailable at the time of surgical decision-making and may lie on the causal pathway between tumor biology and oncological outcomes. Accordingly, these results should be interpreted as complementary to, rather than replacing, the prespecified primary multivariable analysis.

Supplementary Table S2. Baseline characteristics of STAS-positive patients undergoing lobectomy or sublobar resection.

VARIABLE		STAS+ LOBAR (71)	STAS+ SUBLOBAR (40)	P-VALUE
**AGE, y, mean (SD)**		68.8 (9.8)	70.9 (9.8)	0.2
**SEX, Male, n (%)**		31 (43.7%)	20 (50.0%)	0.5
**ASA, mean (SD)**		1.6 (0.61)	1.8 (0.62)	0.1
**BMI, mean (SD)**		25.4 (4.2)	24.7 (4.0)	0.4
**FEV1, mean (SD)**		92.6 (20.4)	87.3 (22.8)	0.2
**DLCO, mean (SD)**		73.6 (15.0)	66.1 (16.1)	0.01
**CAD, yes, n (%)**		6 (8.4%)	8 (20.0%)	0.07
**AD, yes, n (%)**		5 (7.4%)	5 (12.5%)	0.3
**CVD, yes, n (%)**		4 (4.2%)	4 (10%)	0.2
**CKD, yes, n (%)**		10 (14.1%)	7 (17.5%)	0.6
**CLD, yes, n (%)**		9 (12.7%)	10 (25%)	0.1
**DIA, yes, n (%)**		14 (19.7%)	5 (12.5%)	0.3
**PND, yes, n (%)**		18 (25.3%)	10 (25%)	0.9
**pT, n (%)**				0.1
	**1**	39 (54.9%)	28 (70%)	
	**2**	20 (28.2%)	11 (27.5%)	
	**3**	9 (12.7%)	0 (0%)	
	**4**	3 (4.2%)	1 (2.5%)	
**pN, n (%)**				0.3

**0**	48 (67.6%)	32 (80.0%)
**1**	11 (15.5%)	5 (12.5%)
**2**	12 (16.9%)	3 (7.5%)

Values are mean (SD), No. (%), or as otherwise indicated. ASA: American Society of Anesthesiologists, BMI: body mass index, FEV1: forced expiratory volume in 1 second, DLCO: carbon monoxide lung diffusion capacity, CAD: coronary artery disease, AD: arterial vascular disease, CVD: cerebrovascular disease, CKD: chronic kidney disease, CLD: chronic liver disease, DIA: diabetes, PND: previous neoplastic disease, pT: pathologic T stage, pN: pathologic N stage

Footnote

Among the baseline variables examined, only DLCO differed significantly between patients undergoing lobectomy and those undergoing sublobar resection.
